# Self-medication and its risk factors among women before and during pregnancy

**DOI:** 10.11604/pamj.2017.27.183.10030

**Published:** 2017-07-07

**Authors:** Hossein Ebrahimi, Giti Atashsokhan, Farzaneh Amanpour, Azam Hamidzadeh

**Affiliations:** 1 Center for Health Related Social and Behavioral Sciences Research, Shahroud University of Medical Sciences, Shahroud, Iran; 2 Department of Midwifery, School of Nursing & Midwifery, Shahroud University of Medical Sciences, Shahroud, Iran; 3 Department of Epidemiology and Biostatistics, School of Public Health, Shahroud University of Medical Sciences, Shahroud, Iran

**Keywords:** Self medication, risk factors, pregnancy

## Abstract

**Introduction:**

Self-medication can cause significant challenges for the individuals and community, especially in women during pregnancy. This study was aimed to compare the prevalence of self-medication before and during pregnancy among women in Iran.

**Methods:**

in this cross-sectional study, a total of 384 pregnant women were evaluated for the prevalence of self-medication and its associated factors before and during pregnancy. Stratified random sampling was used as the sampling method. Descriptive statistics and chi-square and logistic regression tests were used for statistical analysis of data.

**Results:**

The results showed that the prevalence of self-medication, in women who had become ill at least once, was 63.9% before pregnancy and 43.5% and during pregnancy. Variables such as lack of insurance, high school education and not having a child increased odds ratio of self-medication before pregnancy, while the variables of lack of insurance, not having a child or fewer number of children and no history of abortion increased the odds ratio of self-medication during pregnancy.

**Conclusion:**

Although the prevalence of self-medication during pregnancy was less than that before pregnancy, but this prevalence during pregnancy was still significant. Therefore, it seems necessary to provide public trainings for all women of reproductive age and train them about the dangers and side effects of self-medication.

## Introduction

Self-medication is an important part of daily self-care and one of the vital issues of health care systems [1]. Self-medication is a serious economic, social and health problem throughout the world, including Iran [2]. Some of the reasons for the increased rate of self-medication are the followings: the urge to self-care, feeling sympathy for the family members during illness, lack of health care services, poverty, ignorance, misconceptions, extensive advertisements on drugs and the availability of drugs in places other than pharmacies [3]. Given the current global economic downturn and due to the difficulties to meet the health care needs of the people, many countries are facing serious health challenges [4]. Such a condition in developing countries often motivates most of the people to practice self-medication using different types of materials and plants so that to meet their medical needs [5]. The prevalence of self-medication widely varies in different countries (6%-73%) [6–10]. The prevalence of self-medication in Iran is reported from 76.6% to 83% [2, 11, 12]. The pattern of self-treatment varies in different communities and is affected by several factors such as age, sex, income, expenses, self-care orientation, education level, medical knowledge, satisfaction and people's perception of disease [1]. In most developing countries where the health systems are not properly qualified, there is a high possibility of self-medication in pregnant women [13]. The use of medication during pregnancy still remains a medical challenge [14]. It is estimated that 10% or more than 10% of birth defects are caused due to the exposure of pregnant women to drugs [15]. Many studies have shown that drug use and self-medication during pregnancy may affect fetal health [16, 17]. The prevalence of self-medication during pregnancy varies in Iran and other countries (5%-92.6%) [13, 18–23]. Given the above mentioned facts and since we did not find any study comparing the prevalence of self-medication before and during pregnancy, this study was aimed to determine the prevalence of self-medication and its determinants among the pregnant women in Iran.

## Methods

In this cross-sectional study, a total of 384 pregnant women were evaluated for the prevalence of self-medication and its associated factors before and during pregnancy. The samples were selected via stratified random sampling method. The study was approved by the ethics committee of Shahroud University of medical sciences (project number: 87/930. Approval Date: 2015/4/8). Pregnancy was the inclusion criterion; the exclusion criteria were long-term use of drugs or history of any physical or mental illness. To obtain the required data, we used a form for collecting demographic data and a checklist for obtaining data on history of diseases and self-medication before and during pregnancy. It is worth mentioning that the question about the menstrual disorders was only used for the period before pregnancy, while the question about the problems during pregnancy was only used for the period during the pregnancy. All individuals who used drugs before or during pregnancy without a prescription by a physician were identified as cases practicing self-medication. The data collection was carried out from the beginning of October until the end of January 2014. After obtaining informed consent, the women were enrolled in the study. The selected women, in their last visit for prenatal care, completed the checklist designed for the evaluation of self-medication before and during pregnancy. Self-medication before pregnancy was evaluated during the six months before gestation. To calculate the sample size we considered α= 5%, self-medication ratio of 50% and an accuracy of 95%; the final self sample size was 384. To analyze the data we used descriptive statistics and chi-square and logistic regression tests were used for statistical analysis.

## Results

According to the results, the mean age of the women studied was 26.33±4.60 years, the mean number of children was 0.75, the mean height of women was 160.48 ± 5.33, the mean body weight was 60.31±0.14, the mean BMI was 23.42 ± 3.81, the mean systolic blood pressure was 116.92 ± 8.75 and the mean diastolic blood pressure was 74.64 ± 8.32, respectively. Table 1 shows distribution absolute and relative frequency of study subjects in terms of employment status, education level and insurance status.

**Table 1:** Distribution absolute and relative frequency of study subjects in terms of employment status, education level and insurance status

Variable		Number	%
Job	Housewife	320	16.7
	Employed	64	83.3
Education	Illiterate	4	1.0
	Elementary	22	5.7
	Guidance	45	11.7
	Diploma	165	43.0
	Higher Education	148	38.5
Insurance	Yes	178	46.4
	No	206	53.6

53.4% of the participants had become ill at least once prior to the pregnancy of whom 63.9% had a history of self-medication. Of all the women surveyed, a total of 131 pregnant women (34.1%) reported taking at least one type of drug for self-medication. Moreover, 49.1% of the participants in the study had become ill at least once during pregnancy, of whom 43.5% used at least one drug for self-medication; of all the women studied, 78 patients (20.3%) reported taking at least one drug for self-medication during pregnancy. The results of Chi-square test indicated that self-medication during pregnancy was significantly lower than that before pregnancy (P < 0.001) (Table 2). Table 3 presents the results of logistic regression analysis which was conducted to estimate the odds ratio (OR) of self-medication before pregnancy and its risk factors. It compared the women who practiced self-medication compared with the women who did not practice self-medication before getting pregnant.

**Table 2:** Distribution absolute and relative frequency of self-medication before and during pregnancy

Self-medication		During pregnancy		Total
		No: N (%)	Yes: N (%)	N (%)
Before Pregnancy	No: N (%)	226 (58.9)	27 (7)	253 (65.9)
	Yes: N (%)	80 (20.8)	51 (13.3)	131 (34.1)
Total: N (%)		306 (79.7)	78 (20.3)	384 (100)

**Table 3:** The role of different independent variables on self-medication before pregnancy

Independent variable		Adjusted Odds Ratio (95% CI)	P value
Age		1.03 (0.93-1.14)	0.562
Job	Housewife	Reference	
	Employed	2.31 (0.87-5.96)	0.083
Insurance	Yes	Reference	
	No	2.03 (1.00- 4.10)	0.049
Education	College	Reference	
	High School	2.96 (1.33- 6.61)	0.008
	Primary	1.89 (0.66- 5.40)	0.233
Income	>7000000	Reference	
	≤7000000	0.65 (0.30- 1.39)	0.264
Child	≥2	Reference	
	1	1.33 (0.50- 3.53)	0.568
	0	3.18 (1.04- 9.73)	0.042
abortion	Yes	Reference	
	No	1.88 (0.75- 4.73)	0.178

The results showed that the variables of insurance, education level and number of children had an impact on self-medication before pregnancy, so that the odds ratio of self-medication among those without any insurance was almost twice as much as that in women with insurance. The odds ratio of self-medication in women with secondary education (Group 2) was almost three times higher than that in people with high education level (group 3). In addition, the odds ratio of self-medication in women without a child was almost three times higher than that in those with two children or more.

Table 4 presents the results of logistic regression analysis which was conducted to estimate OR of self-medication during pregnancy and its risk factors. It compared the women who practiced self-medication compared with the women who did not practice self-medication during pregnancy. The results showed that the factors of insurance, number of children and history of abortion had an impact on self-medication in women who become ill during pregnancy. Thus, the odds ratio of self-medication was higher in people without insurance than those with insurance. The odds ratio of self-medication in women without a child and with only one child was 6.8 and 5.4 times higher than those with more than two children. In addition, the odds ration of self-medication in women without a history of abortion was 2.8 times higher than those with a history of abortion.

**Table 4:** The role of different independent variables on self-medication during pregnancy

Independent variable		Adjusted Odds Ratio (95% CI)	P value
Age		1.03 (0.92- 1.14)	0.630
Job	Housewife	Reference	
	Employed	1.17 (0.41- 3.25)	0.783
Insurance	Yes	Reference	0.014
	No	0.39 (0.18- 0.83)	
Education	College	Reference	
	High School	1.21 (0.51- 2.87)	0.668
	Primary	1.38 (0.42- 4.55)	0.594
Income	>7000000	Reference	
	≤7000000	0.84 (0.38- 1.88)	0.676
Child	≥2	Reference	
	1	5.42 (1.44- 20.40)	0.012
	0	6.86 (1.53- 30.65)	0.012
abortion	Yes	Reference	
	No	2.85 (1.02- 7.91)	0.045

Table 5 presents the results of logistic regression analysis which was conducted to estimate OR of self-medication before and during pregnancy and its risk factors. It compared the women who practiced self-medication compared with the women who did not practice self-medication before and during pregnancy. The results showed that individuals who become ill both before and during pregnancy, the variables of insurance, number of children, history of abortion and drug use before pregnancy had an effect on drug use during pregnancy. Accordingly, people without insurance had lower odds ratio of self-medication during pregnancy than those with insurance. The odds ratio of self-medication in women without a child and with only one child was higher than those with more than two children. Women with no history of abortion had higher odds ratio of self-medication during pregnancy than those who had a history of abortion. In addition, people who took medications before pregnancy had a higher chance of self-medication during pregnancy compared with those who had not a history of self-medication before pregnancy.

**Table 5:** The role of different independent variables on self-medication before and during pregnancy

Independent variable		Adjusted Odds Ratio (95% CI)	P value
Age		1.04 (0.89 - 1.21)	0.597
Job	Housewife	Reference	
	Employed	0.94 (0.24 - 3.63)	0.930
Insurance	Yes	Reference	
	No	0.17 (0.06 - 0.53)	0.002
			
Education	College	Reference	
	High School	1.15 (0.35 - 3.74)	0.819
	Primary	1.79 (0.39 - 8.29)	0.455
Income	>700	Reference	
	≤700	0.81 (0.27- 2.39)	0.702
Child	≥2	Reference	
	1	11.45 (2.29 - 57.17)	0.003
	0	9.91 (1.55 - 63.42)	0.015
abortion	Yes	Reference	
	No	5.62 (1.27 - 24.88)	0.023
self-medication before pregnancy	No	Reference	
	Yes	5.11 (1.69- 15.39)	0.004

## Discussion

The results of this study showed that 53.4% of the participants had become ill at least once prior to the pregnancy of whom 63.9% had a history of self-medication. Of all the women surveyed, a total of 131 pregnant women (34.1%) reported taking at least one type of drug for self-medication. The results of previous studies also show that the rate of self-medication is high both in developed and developing countries. For example, according to different reports the prevalence of self-medication is more than 68% in European countries [24], 59% in Nepal (10), 76% in Pakistan [25] and from about 76.6% to 83% in Iran [2, 11, 12]. Easy purchase of drugs without prescription, easy access to drugs and prescribing excessive drugs for patients in previous visits are among the factors that could lead to the storage of drugs at home and consequently lead to self-medication. Self storage of drugs at home not only increases the chance for self-medication, but also raises some other issues such as the proper storage of drugs, expiration date, the possibility of errors in the use of drugs, and other people's access to drugs [24]. In this study, the prevalence of self-medication during pregnancy was 20.3%. This rate of self-medication is consistent with the results of a study in Ethiopia which reported a rate of 20.1% (20), but it is higher than the prevalence rates reported by other studies in Iran [26], Ethiopia [27] and Peru [28] where the prevalence rates are 12%, 12.4%, 10.5% and 8.8% respectively. Nevertheless, the reported prevalence rate in our study was lower than that reported in a study in Egypt which was 86% [29]. In line with the results of Kebede et al (2009), it seems that mild disease and fear of side effects of medication on the fetus are the main reasons for the reduction of self-medication during pregnancy [27].

According to the results of this study, the rate of self-medication during pregnancy, compared with the time before pregnancy, decreased and the difference was statistically significant. In line with our findings, according to the result of a study in Ethiopia, the prevalence of self-medication before pregnancy was 63.7%, while it was 20.1% during pregnancy [20]. Various studies have shown that women are plainly interested in self-medication and they usually practice self-medication to treat problems such as dysmenorrhea, to relieve symptoms of menopause, menstrual disorders and to prevent osteoporosis, however, during pregnancy due to fear of side effects on fetus and embryonic malformations, pregnant women are more cautious about medication [27]. Odalovic et al (2013) reported that women who had a history of at least one delivery are less likely to take medications during pregnancy because they are more alert and aware of the potentially harmful effects on fetus [30]. But the result of a study in Saudi Arabia is inconsistent with the results of our study; according to results of the mentioned study, most women believed that drug consumption during pregnancy was not harmful however, they used drugs with caution [31]. It seems that social and individual factors such as occupation and level of education have a significant impact on the attitudes and beliefs of pregnant women about self-medication and they may be the possible reason for the discrepancy in the results.

In this study, the odds ratio of self-medication in terms of insurance coverage was significantly different among the participants before pregnancy. Accordingly, odds ratio of self-medication among people without health insurance was about twice as much as that among women with insurance. This finding is in line with the results of a study conducted in Ardebil [32]. The researchers of the mentioned study reported that some factors such as lack of insurance and financial difficulties were among the factors encouraging people to self-medicate. It seems that for people who do not have insurance, the cost of treating common diseases such as headaches and colds via self-medication is lower than the cost of visits to physicians, because in the latter case, the patient not only must pay for the medications but also for the visit as well. Hence, the patent would prefer self-medication. Low drug cost and lack of insurance coverage could affect and lead to unreasonable drug consumption. Many families have a drug box at their home which they use for treating different diseases.

Based on the results of this study, before pregnancy, women with high school education self-medicated three times more than women with high education. The results of studies conducted in China [33] and India [34–36] indicate that people with higher levels of education are more prone to self-medication than those with a lower education level; it is not in line with our results. It seems that higher rates of self-medication in people with higher education in the mentioned studies are due to their ability to learn from brochures and repeat prescriptions when they are affected by the same disease. Another possible reason for this discrepancy can be due to different levels of participants' access to health care providers and their lower financial ability to pay for medical costs. Contrary to our findings, the results of a study by Afshary et al (2015) showed that the highest prevalence of self-medication in pregnant women was observed among those with high school and academic education. According to the mentioned researchers, this is due to the fact that these women can get enough information from drug brochures. The results of the study indicated that the prevalence of self-medication in pregnant women was more common among those with an education level less than a high school; it might be attributed to lack of financial capability to pay for medical expenses [26]. According to the results of this research, there was a statistically significant relationship between the number of children and self-medication before pregnancy, so that the odds ratio of self-medication among people without children was three times more than those with more than two children. Results of a study in Brazil showed that the rate of self-medication among people with children was less than that among those without a child; in other words, having a child was a protective factor against self-medication [37]. It seems that previous history of childbirth and concerns about bearing children with congenital anomalies are among the factors which reduce the possibility of self-medication among women with more children.

Although having insurance increased the chance of self-medication during pregnancy, Ghanei et al (2013) found no statistically significant relationship between insurance coverage and self-medication among pregnant women [38]. However, Shamsi et al (2010) reported that the main causes of self-medication in pregnant women were neglecting the impact of the diseases (58%) and lack of insurance coverage (56%). They also reported that people practiced self-medication because they did not pay for visits, obtained necessary medicines by their own and used their previously prescribed medications [39]. It seems that in this study, women with insurance practiced self-medication due to the following factors: reuse of previously prescribed medications, storing medication at home and the high cost of visits to physicians. In this study, the OR of self-medication during pregnancy among women with no children or with one child, respectively, was 6.86 and 5.42 times more than that among women with two or more children in this study. Afshary et al (2015) reported no statistically significant relationship between the number of pregnancies and childbirth with self-medication [26]. In Shamsi et al's study (2010), the highest frequency of self-medication was observed among women with four or more children; the higher rate of self-medication among the mentioned group was attributed to their inability to pay for treatment costs, more cases of childbirth and consequently more experience and knowledge of pregnant women about prescribed drugs [39]. It seems that the lower rate of self-medication reported in this study can be attributed to their higher levels of experiences and knowledge about low-risk pregnancy. Inconsistent with the findings of our study, Guerra et al (2008) reported a positive relationship between self-medication and having more children. The researchers did not find any reason for this relationship in the medical literature; however, they said that the higher rates of self-medication among multiparous women might be due to their previous experiences of self-treatment which might led them to consider such a practice to be harmless. Because of such safe experiences, the mentioned group of women continued self-medication during pregnancy [28].

In this study, the odds ratio of self-medication during pregnancy among women without a history of abortion was 2.85 times more than that among women who had experienced at least one abortion. Mohammad et al (2013) did not observe any statistically significant relationship between self-medication and previous history of abortion among pregnant women in Ethiopia [40]. In our review of available medical literature, we did not find any data about the relationship between abortion and self-medication. However, it seems that, patients with a history of abortion are less prone to self-medication because they may believe that self-medication may lead to abortion; hence, this group of women are more sensitive toward their pregnancy status and are less likely to self-medicate. Our research showed that people who had used drugs before pregnancy, compared with those who did not take medications before pregnancy, had a higher odds ratio of self-medication during pregnancy. According to the results of Mohammad et al's study (2013) the continuation of self-medication during pregnancy is due to the lack of proper consultation by health care providers; the researchers concluded that in the absence of medical supervision during pregnancy, the pregnant women continue taking drugs [40]. On the other hand, based on the results of a study on motivational factors for self-medication during pregnancy, the most important factors were: not paying enough attention to the disease and feeling no need for medical care services [29].

## Conclusion

Despite the decrease in the prevalence of self-medication during pregnancy, compared with the time before pregnancy, the prevalence of self-medication during pregnancy was still significant in this study. Therefore, it seems necessary to provide public trainings for all women of reproductive age and train them about the risks and side effects of self-medication. In addition, to promote the health status of people in the community and to increase their knowledge, such training programs must be designed in health care systems.

### What is known about this topic

- Today, self-medication is now one of the greatest healths, economic and social problems in different societies, including Iran;
- Self-medication during pregnancy is associated with adverse effects and lack of knowledge and little information of mothers about use of drugs, could be harmful for the family and society;
- Despite the increase knowledge about the use of drugs during pregnancy in recent years there is increasing evidence that self-medications among pregnant women are common.

### What this study adds

- Although the prevalence of self-medication during pregnancy was less than before pregnancy, but this study shows that self-medication is common among pregnant women in our environment;
- The variables that increased the odds ratio of self-medication, before and during pregnancy, were different;
- Adequate education of pregnant women during antenatal clinics on the potential danger of self-medication is necessary.

## Competing interests

The authors declare no competing interest.

## References

[1] Almasdy D, Sharrif A (2011). Self-medication practice with nonprescription medication among university students: a review of the literature. Archives of Pharmacy Practice..

[2] Karimy M, Heidarnia A, Ghofrani F (2011). Factors influencing self-medication among elderly urban centers in Zarandieh based on health belief model. Arak University of Medical Sciences Journal..

[3] Ahmad A, Patel I, Mohanta GP, Balkrishnan R (2014). Evaluation of self medication practices in rural area of town Sahaswan at Northern India. Annals of Medical and Health Sciences Research..

[4] Irvine L, Flynn RW, Libby G, Crombie IK, Evans JM (2010). Drugs dispensed in primary care during pregnancy: a record-linkage analysis in Tayside, Scotland. Drug safety..

[5] Rohra DK, Das N, Azam SI, Solangi NA, Memon Z, Shaikh AM (2008). Drug-prescribing patterns during pregnancy in the tertiary care hospitals of Pakistan: a cross sectional study. BMC pregnancy and childbirth..

[6] Bamgboye EA, Amoran OE, Yusuf OB (2006). Self medication practices among workers in a tertiary hospital in Nigeria. African journal of medicine and medical sciences..

[7] Corrêa-Fissmer M, Mendonça MG, Martins AH, Galato D (2014). Prevalence of self-medication for skin diseases: a systematic review. Anais Brasileiros de Dermatologia..

[8] Loyola de Filho AI, Uchoa E, Guerra HL, Firmo JO, Lima-Costa MF (2002). Prevalência e fatores associados à automedicação: resultados do projeto Bambuí. Rev Saúde Pública..

[9] Gutema GB, Gadisa DA, Kidanemariam ZA, Berhe DF, Berhe AH, Hadera MG (2011). Self-medication practices among health sciences students: the case of Mekelle University. Journal of Applied Pharmaceutical Science..

[10] Shankar P, Partha P, Shenoy N (2002). Self-medication and non-doctor prescription practices in Pokhara valley, Western Nepal: a questionnaire-based study. BMC family practice..

[11] Jafari F, Khatony A, Rahmani E (2015). Prevalence of self-medication among the elderly in Kermanshah-Iran. Global Journal of Health Science..

[12] Sarahroodi S, Maleki-Jamshid A, Sawalha AF, Mikaili P, Safaeian L (2012). Pattern of self-medication with analgesics among Iranian University students in central Iran. Journal of Family & Community Medicine..

[13] Abasiubong F, Bassey EA, Udobang JA, Akinbami OS, Udoh SB, Idung AU (2012). Self-medication: potential risks and hazards among pregnant women in Uyo, Nigeria. The Pan African medical journal..

[14] Araújo DD, Leal MM, Santos EJV, Leal LB (2013). Consumption of medicines in high-risk pregnancy: evaluation of determinants related to the use of prescription drugs and self-medication. Brazilian Journal of Pharmaceutical Sciences..

[15] Nakamura MU, Kulay Junior L, Pasquale M (2008). Uso de fármacos na gravidez: benefício e custo. Rev bras ginecol obstet..

[16] Chiriboga CA (2003). Fetal alcohol and drug effects. The neurologist..

[17] Szymanowski K, Chmaj-Wierzchowska K, Florek E, Opala T (2006). Influence of tobacco smoking to development of the fetus, newborn and child: a review. Przeglad lekarski..

[18] Bagheri A, Abbaszadeh F (2014). Self-medication and supplement use by pregnant women in Kashan rural and urban areas. Journal of Mazandaran University of Medical Sciences (JMUMS)..

[19] Baghianimoghadam MH, Mojahed S, Baghianimoghadam M, Yousefi N, Zolghadr R (2013). Attitude and practice of pregnant women regarding self-medication in Yazd, Iran. Archives of Iranian medicine..

[20] Befekadu A, Dhekama NH, Mohammed MA (2014). Self-medication and contributing factors among pregnant women attending antenatal care in Ethiopia: The Case of Jimma University Specialized Hospital. Medicine Science..

[21] Emmanuel AE, Achema G, Afoi BB, Maroof R (2014). Self medication practice among pregnant women attending antenatal clinic in selected hospitals in Jos. International Journal of Nursing and Health Science..

[22] Glover DD, Amonkar M, Rybeck BF, Tracy TS (2003). Prescription, over-the-counter and herbal medicine use in a rural, obstetric population. American Journal of Obstetrics and Gynecology..

[23] Liao S, Luo B, Feng X, Yin Y, Yang Y, Jing W (2015). Substance use and self-medication during pregnancy and associations with socio-demographic data: a cross-sectional survey. International Journal of Nursing Sciences..

[24] Bretagne JF, Richard-Molard B, Honnorat C, Caekaert A, Barthelemy P (2006). Gastroesophageal reflux in the French general population: national survey of 8000 adults. Presse medicale, Paris, France. 1983..

[25] Zafar SN, Syed R, Waqar S, Zubairi AJ, Vaqar T, Shaikh M (2008). Self-medication amongst university students of Karachi: prevalence, knowledge and attitudes. JPMA The Journal of the Pakistan Medical Association..

[26] Afshary P, Mohammadi S, Najar S, Pajohideh Z, Tabesh H (2015). Prevalence and causes of self-medication in pregnant women referring to health centers in Southern of Iran. International Journal of Pharmaceutical Sciences and Research..

[27] Deshpande SG, Tiwari R (1997). Self medication: a growing concern. Indian journal of medical sciences..

[28] Guerra GC, da Silva AQ, Franca LB, Assuncao PM, Cabral RX, Ferreira AA (2008). Drug use during pregnancy in Natal, Brazil. Rev Bras Ginecol Obstet..

[29] Garofalo L, Di Giuseppe G, Angelillo IF (2015). Self-medication practices among parents in Italy. Bio Med research international..

[30] Odalovic M, Vezmar Kovacevic S, Nordeng H, Ilic K, Sabo A, Tasic L (2013). Predictors of the use of medications before and during pregnancy. International journal of clinical pharmacy..

[31] Zaki NM, Albarraq AA (2014). Use, attitudes and knowledge of medications among pregnant women: a Saudi study. Saudi pharmaceutical journal: SPJ: the official publication of the Saudi Pharmaceutical Society..

[32] Amani F, Mohammadi S, Shaker A, Shahbazzadegan S (2011). Study of arbitrary drug use among students in Universities of Ardabil City in 2010. Journal of Ardabil University of Medical Sciences..

[33] Yuefeng L, Keqin R, Xiaowei R (2012). Use of and factors associated with self-treatment in China. BMC public health..

[34] Kaushal J, Gupta MC, Jindal P, Verma S (2012). Self-medication patterns and drug use behavior in housewives belonging to the middle income group in a city in northern India. Indian journal of community medicine: official publication of Indian association of preventive & social medicine..

[35] Selvaraj K, Kumar SG, Ramalingam A (2014). Prevalence of self-medication practices and its associated factors in urban Puducherry, India. Perspectives in clinical research..

[36] Shveta S, Jagmohan S (2011). A study of self medication pattern in Punjab. Indian Journal of Pharmacy Practice..

[37] Correa da Silva MG, Soares MC, Muccillo-Baisch AL (2012). Self-medication in university students from the city of Rio Grande, Brazil. BMC Public Health..

[38] Ganaeie R, Hemmati Maslakpak M, Baghi V (2013). Self-medication in pregnant women. Journal of research development in nursing & midwifery..

[39] Shamsi M, Bayati A (2010). A survey of the prevalence of self-medication and the factors affecting it in pregnant mothers referring to health centers in arak city, 2009. Pars journal of medical sciences: jahrom medical journal..

[40] Mohammed MA, Ahmed JH, Bushra AW, Aljadhey HS (2013). Medications use among pregnant women in Ethiopia: a cross sectional study. Journal of Applied Pharmaceutical Science..

